# Threatened or Actual Epidemics

**Published:** 1882-09

**Authors:** 


### THREATENED OR ACTUAL EPIDEMICS.

The following circulars contain information concerning the relations of the Marine Hospital Service to the prevalence of contagious and epidemic diseases that should be known to the whole profession :

WASHINGTON, D. C., August 9, 1882.

*To Medical Officers of the Marine Hospital Service and Officers of State and Municipal Boards of Health :*

I am directed by the Secretary of the Treasury to inform you that Congress, at its last session, enacted that:—

"The President of the United States is hereby authorized, in case of a threatened or actual epidemic, to use a sum not exceeding one hundred thousand dollars, out of any money in the treasury not otherwise appropriated, in aid of State and local boards, or otherwise in his discretion, in preventing and suppressing the spread of the same."

He further directs me to inform you that the President has decided to employ this contingent appropriation through the agency of the Treasury Department, and that, in case of a threatened or actual epidemic, immediate action will be taken, upon application from the Governor of a State addressed to the Secretary of the Treasury.

JOHN B. HAMILTON,
*Supervising Surgeon-Gen'l U. S. Marine Hospital Service.*

---

WASHINGTON, D. C., August 11, 1882.

*To Medical Officers of the Marine Hospital Service :*

The attention of all officers is called to the following paragraphs of the regulations governing this service, approved by the Secretary of the Treasury November 10, 1879 :

"*Paragraph* 61.

"Medical officers and acting assistant surgeons of the Marine Hospital Service will inform themselves fully as to the local health laws, and the regulations based thereon and in force at their respective ports and stations, and will comply strictly therewith."

"*Paragraph* 62.

"Medical officers and acting assistant surgeons are, under the direction of the Surpervising Surgeon-General, required to observe and to aid in executing the quarantines and other restraints established by the health laws of any State, and to report forthwith to the said Surgeon-General any important event or fact that may come to their knowledge bearing upon the importation, outbreak, or spread of cholera, yellow fever, small-pox, typhus, or other epidemic disease, at or near their respective stations."

A strict compliance with these paragraphs is enjoined.

JOHN B. HAMILTON,
*Surgeon-General U. S. Marine Hospital Service.*

APPROVED : H. F. FRENCH, *Acting Sec'y of the Treasury.*

